# Human Mammary Epithelial Cells Exhibit a Bimodal Correlated Random Walk Pattern

**DOI:** 10.1371/journal.pone.0009636

**Published:** 2010-03-10

**Authors:** Alka A. Potdar, Junhwan Jeon, Alissa M. Weaver, Vito Quaranta, Peter T. Cummings

**Affiliations:** 1 Department of Chemical and Biomolecular Engineering, Vanderbilt University, Nashville, Tennessee, United States of America; 2 Vanderbilt Integrative Cancer Biology Center, Nashville, Tennessee, United States of America; 3 Department of Cancer Biology, Vanderbilt University Medical Center, Nashville, Tennessee, United States of America; 4 Center for Nanophase Materials Sciences, Oak Ridge National Laboratory, Oak Ridge, Tennessee, United States of America; German Cancer Research Center, Germany

## Abstract

**Background:**

Organisms, at scales ranging from unicellular to mammals, have been known to exhibit foraging behavior described by random walks whose segments confirm to Lévy or exponential distributions. For the first time, we present evidence that single cells (mammary epithelial cells) that exist in multi-cellular organisms (humans) follow a bimodal correlated random walk (BCRW).

**Methodology/Principal Findings:**

Cellular tracks of MCF-10A pBabe, neuN and neuT random migration on 2-D plastic substrates, analyzed using *bimodal analysis*, were found to reveal the BCRW pattern. We find two types of exponentially distributed correlated flights (corresponding to what we refer to as the directional and re-orientation phases) each having its own correlation between move step-lengths within flights. The exponential distribution of flight lengths was confirmed using different analysis methods (logarithmic binning with normalization, survival frequency plots and maximum likelihood estimation).

**Conclusions/Significance:**

Because of the presence of non-uniform turn angle distribution of move step-lengths within a flight and two different types of flights, we propose that the epithelial random walk is a BCRW comprising of two alternating modes with varying degree of correlations, rather than a simple persistent random walk. A BCRW model rather than a simple persistent random walk correctly matches the super-diffusivity in the cell migration paths as indicated by simulations based on the BCRW model.

## Introduction

Cell migration is an important process for a wide range of domains from bacteria to mammals. For prokaryotes (e.g., bacteria), migration is important to locate food sources [1]. Similar goals may apply to unicellular eukaryotes (e.g., *Dictyostelium*). In contrast, in “higher” multi-cellular eukaryotes (e.g., mammals) cell migration is involved in physiological processes as well as pathogenic conditions such as cancer metastasis [2]. Mammalian cell migration is generally thought of as having a “purpose” (such as embryogenesis [3] and immune response [4]) other than locating nutrients and it is believed that these cells follow orders from a higher “programming center”. When these orders are misinterpreted or disregarded, cancer may occur. This paper, instead, is informed by a different premise: namely, that individual cell migration in random motility conditions can be interpreted as a problem of how to efficiently perform a search to locate randomly distributed “target items” (such as nutrients and growth factors) which could only be detected in limited vicinity. This is analogous to animal foraging problem where animals come to adopt an optimal search strategy to locate food.

Random walk theories have been used to model animal displacements to explain optimal foraging, predator-prey relationships, etc. For long-times, animal movements can be modeled as uncorrelated random walks with normal diffusion (mean-squared displacement (MSD), 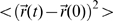 scaling as 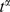 where, 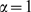) [5], [6], where 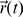 is the position of the animal at time 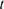 and the average (<>) is over all the members of the population. Anomalous diffusion arises when 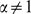, with 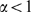 corresponding to sub-diffusive motion, 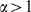 to super-diffusive motion and ‘ballistic motion’ for the case of 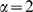. The directional persistence in animal movements has been modeled using correlated random walks or Lévy motion [7]. Correlated random walks have an exponentially decreasing distribution of move step-lengths (distance traveled in one sampling time) [8] and the shape of the turn angle distribution between these move step-lengths controls the directional memory. Lévy motion (Lévy walks or Lévy flights [9], [10], [11] where Lévy walk has a finite mean-squared displacement while a Lévy flight does not) comprises of random walks wherein long flights or steps are separated by shorter jumps. These walks are described by the power-law probability distribution function for the flight or step-length 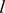, given by 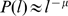, 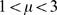 where 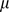 is the ‘Lévy index’. The MSD in Lévy motion always scales as 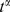 where 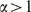 while a correlated random walk eventually loses super-diffusivity reaching normal diffusion once the correlation is lost. Lévy motion converges to Brownian motion for 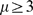.

Lévy motion has been frequently used to model animal displacements in ecology. It has been shown that the efficiency of animal searches incorporating Lévy motion is higher than those using correlated random walks [7] since the chance of returning to the same place with Lévy flights or Lévy walks is less [12], optimizing the predator-prey encounter [13]. Simulations also revealed that for animal foragers feeding on randomly distributed target sites an inverse square power-law distribution of flight lengths in Lévy motion is an optimal solution [12]. Subsequently, experimental studies also reported that organisms ranging from birds [14] to mammals [8] adopt Lévy motions with 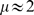. Lévy behavior has been reported in diverse species from marine predators [8], spider monkeys [15], micro-zooplankton [16], soil amoebas [17], freshwater *Hydra* cells [18] and humans [19]; initially, albatrosses were thought to exhibit Lévy behavior [14].

Recently, however, the biological existence of Lévy motion has been questioned and it has been suggested that not all the reported experimental studies follow Lévy behavior [20], [21], [22], [23]. Of particular note is the recent work showing albatross motion is inconsistent with LW behavior [22]. It has been shown that combination random walks such as composite Brownian walks may have a higher search efficiency than Lévy motion and composite, two-search–mode walks (referred to as intermittent search models [24]) can generate patterns that look similar to Lévy motion [20], [24]. However, the intermittent Lévy based models (with Lévy distributed relocation times) outperform the intermittent exponentially distributed ones [25].

It has been suggested in literature that the survival distributions (cumulative frequency of lengths greater than a given length) can correctly identify true Lévy behavior [20], [23], [26]. Also, methods where weights of two competing models (Lévy versus exponential) are calculated (maximum likelihood estimates along with Akaike weight calculations [21], [22] could be useful to identify the true model to describe the observed search patterns.

The persistent random walk model (PRW) [27] (a form of correlated random walk) has often been used to model mammalian cell migration. The PRW model equation involves fitting the experimental mean-squared displacement of cell population with speed and persistence time as two parameters, of which speed is known. We are not aware of any reported attempt to decipher the search pattern of individual mammary epithelial cells in low-cell density conditions in the absence of any biasing cues. Does the random motility of eukaryotic, mammary epithelial cells follow Lévy statistics or not? *A priori*, it might be expected that in the confined *in vitro* conditions the cells may not display the Lévy scale free patterns. Recent studies have indicated that the search strategy of *Dictyostelium* (eukaryotic cell) in the absence of external cues is a persistent cell motion [28], [29]. Van Haastert and coworkers also reported that starved amoeboid cells exhibit correlated random walk food search strategy by extending their run lengths [30]. Our bimodal analysis [31] segregated the motion of mammary epithelial cells into directional or re-orientation phases based on successive angle changes in the nucleus tracks of cells analogous to the segregation of amoeboid tracks into runs (based on splitting pseudopods) and turns (based on *de novo* pseudopods) in the recent work by Van Haastert and coworkers [29], [30], [32]. We report here that the epithelial cell migration paths on 2-D plastic substrates in the absence of any chemo-attractant gradients follow general features of a bimodal correlated random walk model (BCRW). We use the experimental results obtained from application of bimodal analysis (from our previous work, [31]) to model and simulate the random migration search strategy of individual epithelial cells. The data used in this work was obtained by plating cells overnight on tissue culture substrates. Cells were tracked the next day allowing them sufficient time to produce their own extra-cellular matrix on 2-D substrates. The *in vitro* 2-D environments enabled us to collect frequently sampled data (every 30 seconds). This 2-D *in vitro* motion can be expected to be somewhat different from the one in the 3-D *in vivo* conditions (because of the overlaid extra-cellular matrix the frequency of re-orientations can be altered). Nonetheless, characterizing the 2-D motion at high time-resolution can serve as the basis of modeling cellular motion in natural environments.

The BCRW could be thought of as a modified correlated random walk comprising of two alternating modes or “flights” with varying degree of correlations. This is analogous to an intermittent search model having a fast phase oblivious to the presence of any target and a slow responsive search phase to locate the target as described in [24]. In the BCRW model, we refer to flight as the portion of cell path (made of consecutive step lengths taken during successive time steps) comprising either the directional or re-orientation phase (as flagged by our bimodal analysis technique) distinguishing it from a step length taken during a unit time, which in our case was 0.5 minutes. We adopt this terminology from the work by Bartumeus and coworkers [7]. This is not to be confused with the “Levy flight” usage. A directional flight length is the summation of all the consecutive move step-lengths during the directional phase and similarly, a re-orientation flight length is the summation of a series of all the move step-lengths during the re-orientation phase. We define net flight length as the displacement during the given flight.

Some of the salient characteristics of the proposed BCRW are: i) flights follow an exponential distribution; ii) move step-lengths comprising the flight are correlated through turn angles randomly drawn from a distribution such as a Gaussian distribution and iii) move step-lengths within the flight are randomly drawn from a exponential distribution.

## Results and Discussion

**Figure 1:**
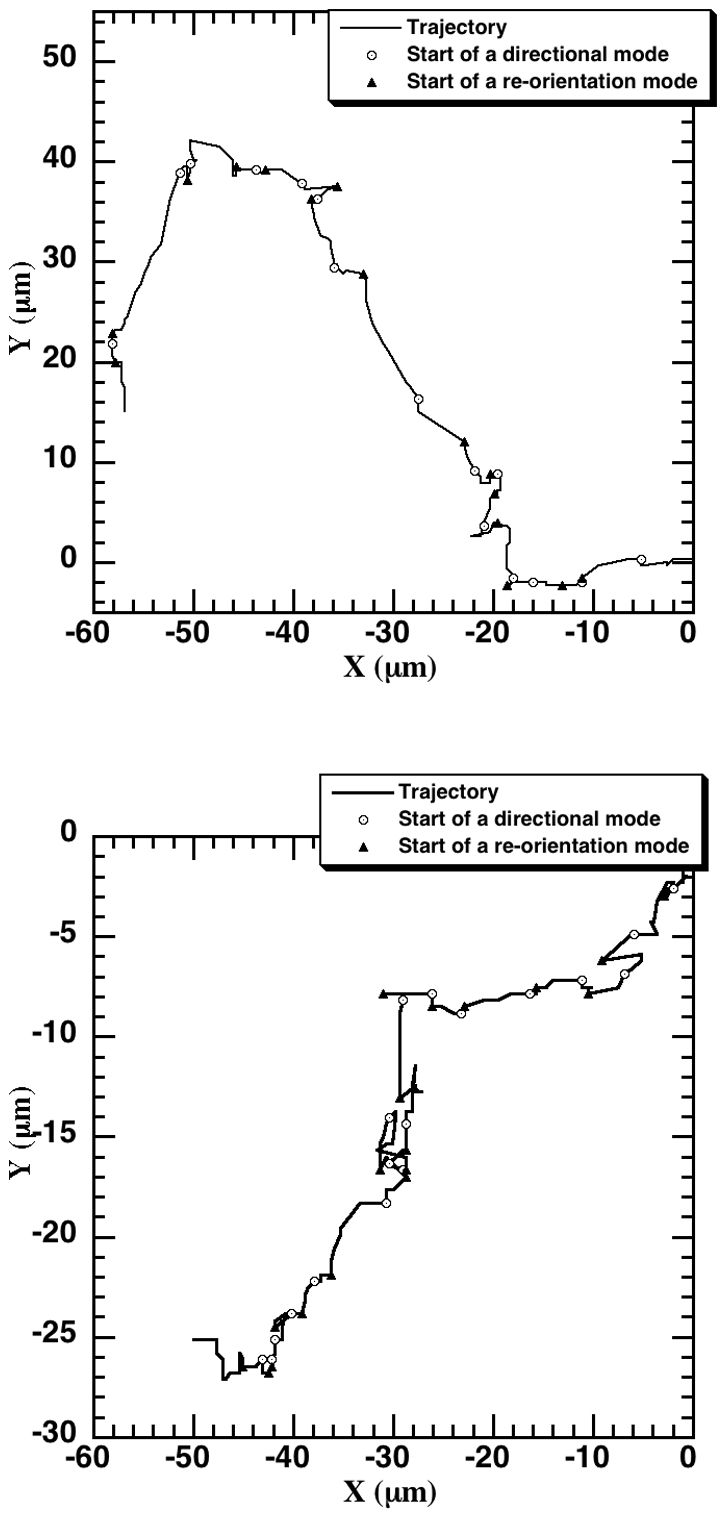
Flagging directional and re-orientation flights using bimodal analysis [31]. An experimental 2-hour neuT (*top*) and pBabe (*bottom*) cell trajectory with the directional and re-orientation flights flagged using bimodal analysis. The cell track starts at origin (0,0) with the start of a directional flight indicated by an open circle and the start of re-orientation denoted by a filled triangle. A directional flight length is the summation of all the consecutive move step-lengths during the directional phase and similarly, a re-orientation flight length is the summation of a series of all the move step-lengths during the re-orientation phase. The net flight length (directional/re-orientation) refers to the net displacement from start to end during the flight.

Recently, we segregated epithelial cell migration tracks into alternating directional and re-orientation modes using our *bimodal analysis* method [31]. Some example trajectories with the directional and re-orientation flights flagged are depicted in Fig. 1 (*top*: neuT [31] and *bottom*: pBabe cell type). The direction changes between consecutive directional flights in epithelial cell random migration was found to be non-instantaneous and the cell was found to spend considerable time in the re-orientation phase. The fundamental idea of a BCRW is applicable for epithelial cell migration, as we have two types of alternating flights (directional and re-orientation flights) with a Gaussian distribution for the turn angles within each flight type and an additional one between two neighboring directional flights. We analyzed the random migration data of MCF-10A pBabe, neuN and neuT human mammary epithelial cells with respect to the distribution of flight lengths (both total distances (referred to as ‘flight’) and net displacement (referred to as a ‘net flight’)), within the above-described general framework of a BCRW to test its applicability to mammalian cell migration.

**Figure 2:**
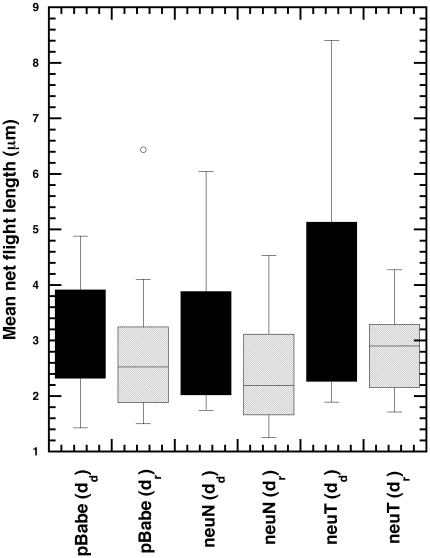
Net flight length during the directional and re-orientation phases for the three cell types. Box plots of the mean net flight lengths during the directional (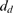) and re-orientation (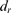) phases for the three cell types (pBabe (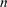 = 15), neuN (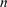 = 15), neuT (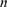 = 12)). The distance traversed in directional flights is more than during re-orientation flights (statistical analysis in Table 1).

We had earlier reported that the mean 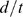 ratios (ratio of displacement to distance for a given flight) in directional phases are higher than those of re-orientation phases [31]. Specifically, we find that the mean displacements (net flight length) during the directional phases (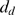) are higher than those during re-orientation phases (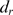) (Table 1) while the mean total distance covered in directional phase (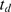) is not statistically different from that during re-orientation phase (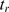) (Table S1). The trends in the net displacements are also implicated in the box plot of the displacements during the different flights for the three cell types (pBabe (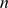 = 15), neuN (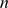 = 15), neuT (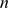 = 12)) (Fig. 2).

**Table 1:** Average net distances covered in directional and re-orientation phases.

		Cell type	
	pBabe	neuN	neuT
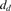 1 ^,^ 3 (micron)	3.09±1.08	3.3±1.33	3.92±1.9
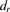 2 ^,^ 3 (micron)	2.79±1.22	2.43±0.97	2.85±0.77
p-values (n^4^)	0.026 (214)	0.001 (187)	0.0326 (169)

Statistical significance to compare between the net distances traveled in the directional (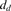) and re-orientation (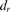) phases using the nonparametric, two-sample Kolmogorov-Smirnov test in MATLAB (kstest2.m). A p-value <0.05 indicates significant difference. A similar analysis was repeated for total distances traveled during the directional flights (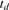) and re-orientation flights (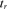) and is shown in Table S1.

1 Mean net distance traveled during directional phase.

2 Mean net distance traveled during re-orientation phase.

3 Mean value reported is the mean of mean values for each cell (pBabe (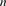 = 15), neuN (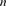 = 15), neuT (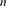 = 12)) while error bars are standard deviation in the means.

4 Sample size for the two groups, which is the number of directional or re-orientation phases.

**Figure 3:**
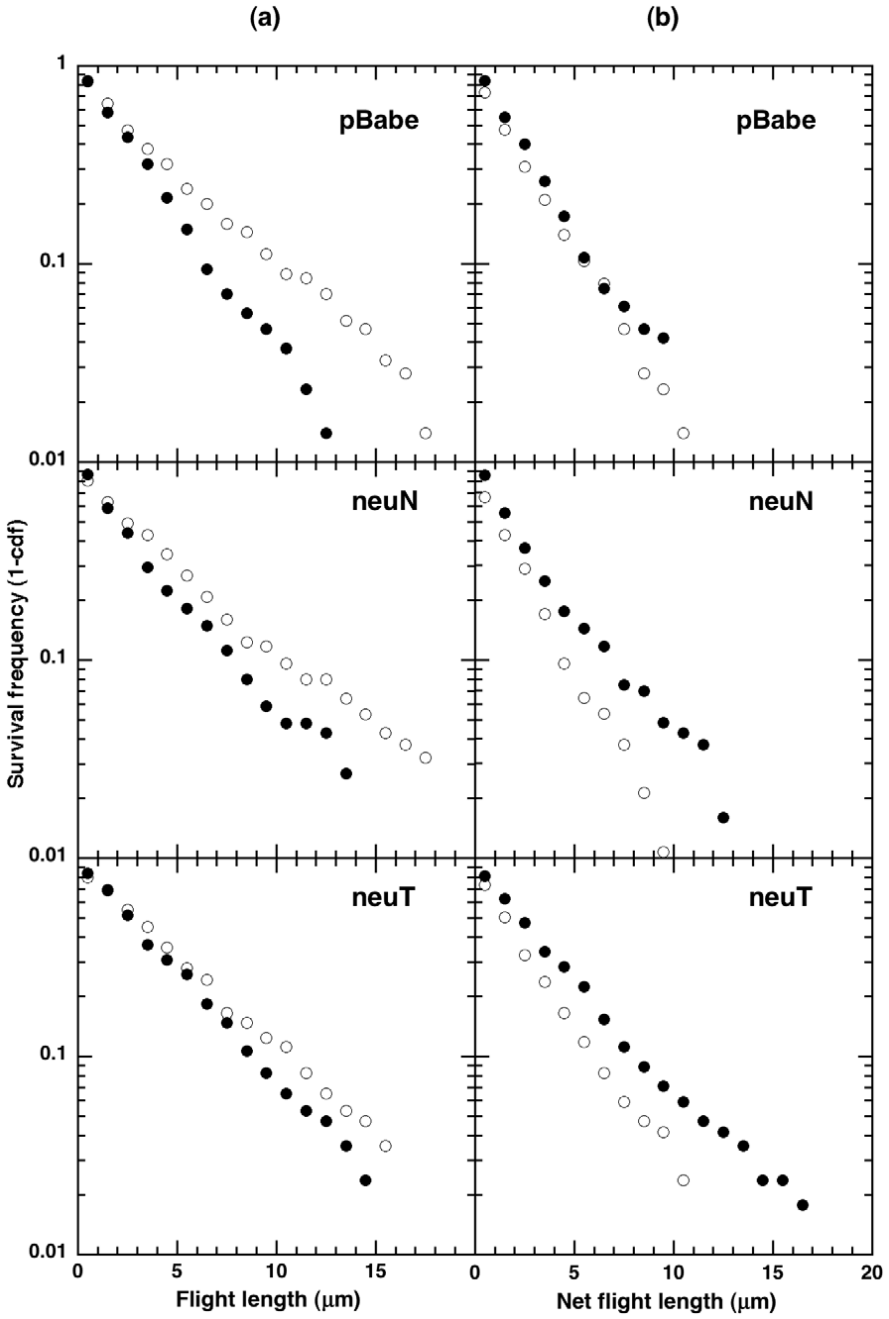
Survival frequency (log-linear) plots for the three cell types in different modes. 3a (*left* panel), the flight length survival frequency plots, filled circles (directional mode) and open circles (re-orientation mode). 3b (*right* panel), the net flight length survival frequency plots, filled circles (directional mode) and open circles (re-orientation mode). The straight-line behavior on the log-linear plots (survival frequency on log scale versus the lengths (flight/net flight) on linear scale) is indicative of exponential distribution of the lengths. The slopes (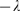 for exponential distribution) along with statistical analysis are shown in Table 2.

In order to test the presence of any Lévy statistics in the flight lengths, we used three different methods (logarithmic binning with normalization, survival frequency plots and maximum likelihood estimation) to analyze the distribution of flights. The first method uses the cumulative distribution/rank frequency plots (flight lengths greater than a given threshold) also referred to as survival distribution [20], [26]. We use the information that the survival distribution would be a straight line on a log-linear scale (probability versus flight length) for an exponential distribution while a true power-law distributed data would be revealed as a straight line on a log-log scale. Likewise, we tested the survival distributions of directional and re-orientation flight distances (referred to as ‘flight length’) and displacements (referred to as ‘net flight length’) for the three cell types. Intriguingly, we found that the flight length survival distributions (both directional and re-orientation) (Fig. 3a, *left* panel) for all cell types on a log-linear scale fitted well with a straight line (statistics, Table 2). Similarly, the net flight lengths (Fig. 3b, *right* panel) also exhibited similar trends (statistics, Table 2). Hence, an exponential model describes the flight lengths/net flight lengths of individual human mammary epithelial cells in random motility conditions.

**Table 2:** Fitting parameters from survival frequency distributions.

	Flights				Net flights			
Cell type (mode)	Slope (− 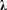 )	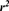	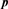 value	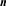	Slope (− 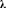 )	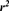	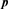 value	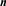
pBabe (Directional)	−0.327	0.993	3.37e-13	214	−0.372	0.978	8.13e-09	214
pBabe (Re-orientation)	−0.221	0.980	5.53e-16	214	−0.387	0.996	1.67e-12	214
neuN (Directional)	−0.255	0.984	3.70e-12	187	−0.293	0.979	1.17e-10	187
neuN (Re-orientation)	−0.187	0.989	3.49e-17	187	−0.437	0.991	1.75e-09	187
neuT (Directional)	−0.249	0.996	1.29e-17	169	−0.239	0.990	1.03e-16	169
neuT (Re-orientation)	−0.208	0.997	3.35e-19	169	−0.372	0.956	3.83e-08	169

Statistical analysis for estimating the fitting parameter for exponential distribution (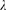) from the survival frequency distributions (regstats.m in MATLAB) with 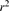 (correlation coefficient) and p-value associated with the slope using t-test indicated.

The slope, 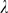 of the straight-line fits (Table 2) on the log-linear survival distributions represents the inverse of the mean flight length. A higher 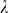 would indicate a smaller mean flight length. The 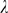 value was found to be higher for the re-orientation net flight compared to the directional net flight consistent with the results in Table 1, giving confidence in the straight-line fits.

**Figure 4:**
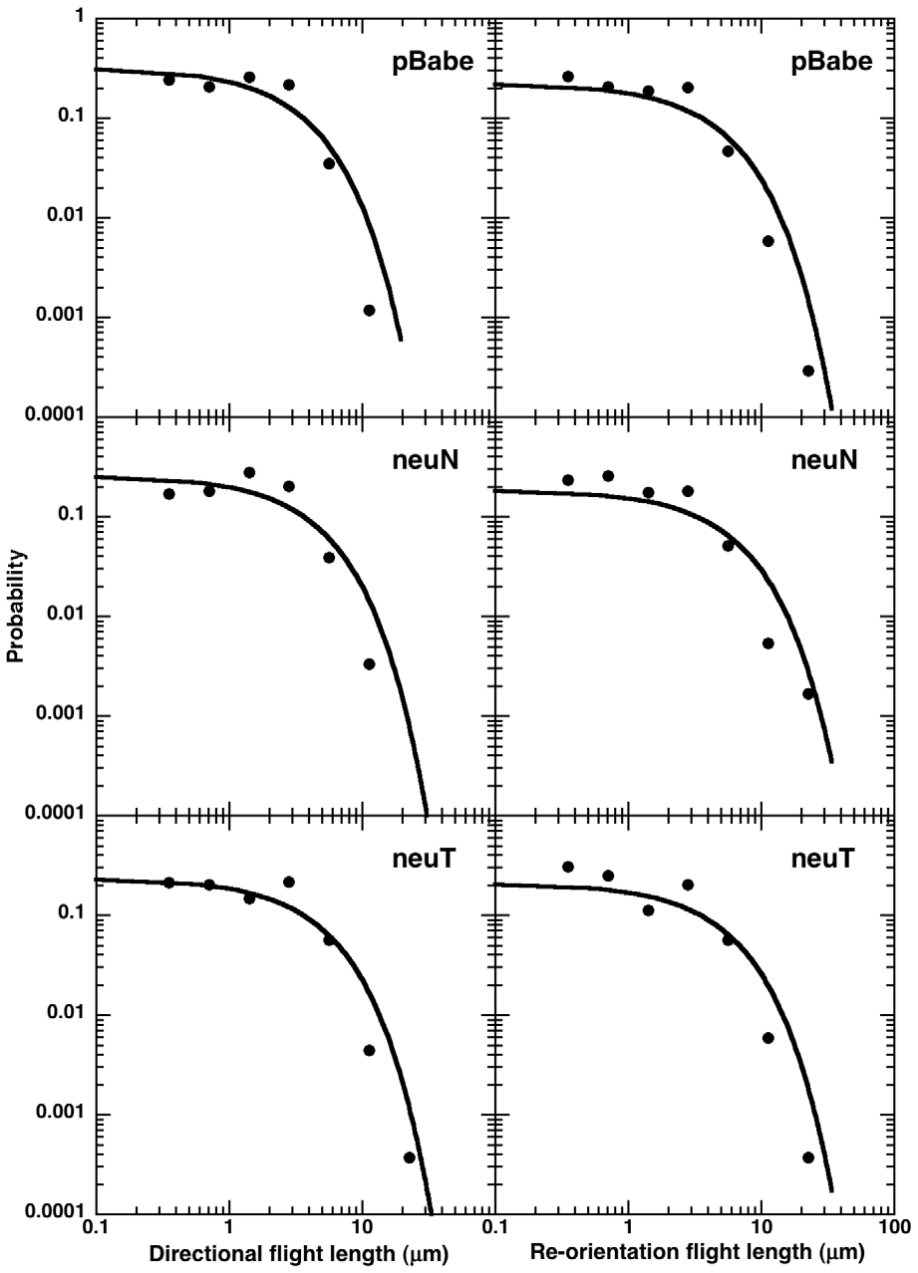
Log-log frequency plots using the logarithmic binning with normalization method along with a fitted exponential function. The logarithmically binned flight length distributions on log-log scale for the three cell types. The directional flight lengths are shown in the left panel while re-orientation flight lengths are on the right. An exponential distribution fitted to the 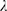 (obtained from corresponding survival distribution) is shown in bold curve in black. The fitted exponential distribution is in good agreement with the experimental data points.

We also used logarithmic binning with normalization method (LBN [23]) to analyze 1140 flights (directional and re-orientation, identified using bimodal analysis [31]) from a total of 42 cells of 3 cell types. The LBN method minimizes errors in identifying Lévy flight behavior, as simple non-normalized frequency linear binning can give erroneous results wrongly identifying Brownian random walks as Lévy [20], [23]. As expected from the results from survival distributions, we do not see the signature power-law linear relationship on log-log scale for both flights (Fig. 4) and net flights (Fig. S1). An exponential distribution fitted to the 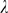 (obtained from survival distributions in Figs. 3a and b), is indicated by the bold, black curve in Figs. 4 and S1. One can see that the experimental data (filled circles) clearly fit the exponential distribution much better than a Lévy model. We also used maximum likelihood estimates and Akaike weights [21], [22] to determine which of the two models (exponential or power law) fit our experimental data (see Text S1; Table S2). The exponential model was favored having higher Akaike weights.

**Figure 5:**
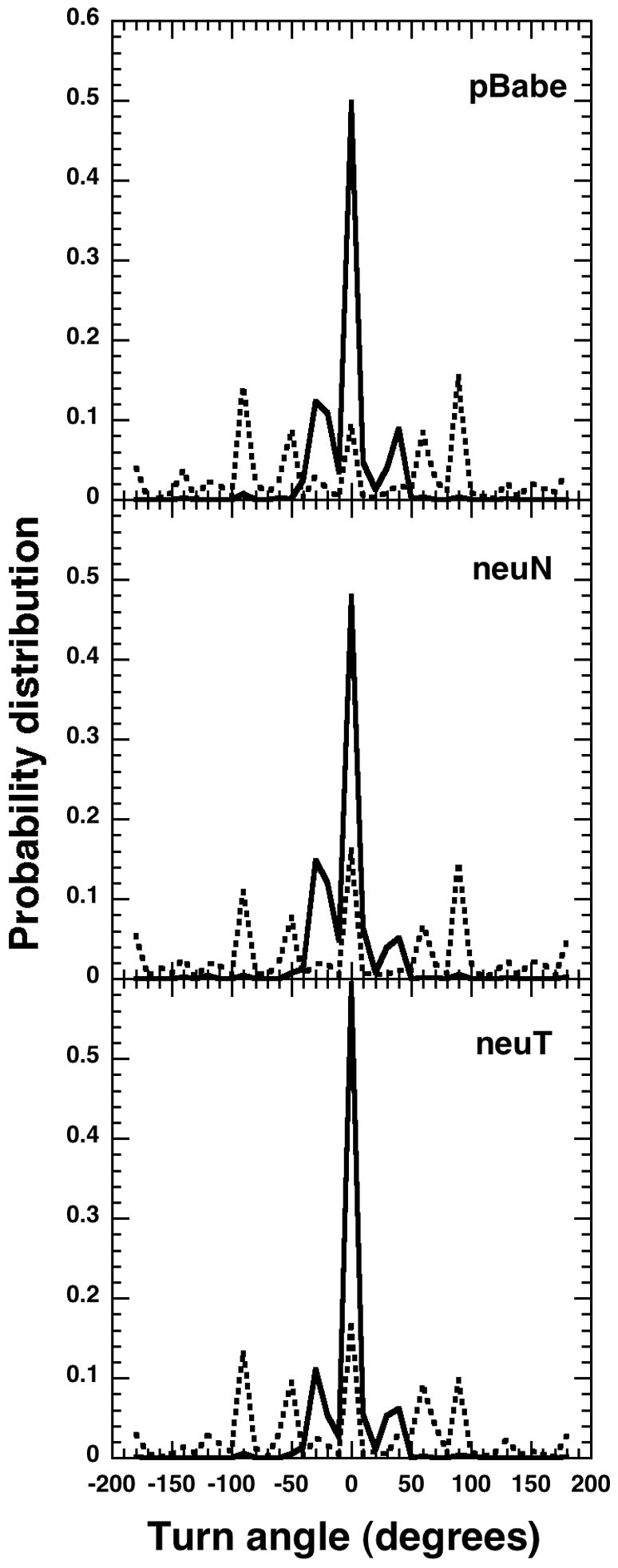
Probability distributions of the turn angles within the directional and re-orientation flights. *Top*, pBabe, *middle*, neuN and *bottom*, neuT cells. The solid line shows the turn angle distribution during directional flights while the broken lines during re-orientation flight. The directional flights display higher persistence compared to the re-orientation flights that have a more flatter turn angle distribution.

**Figure 6:**
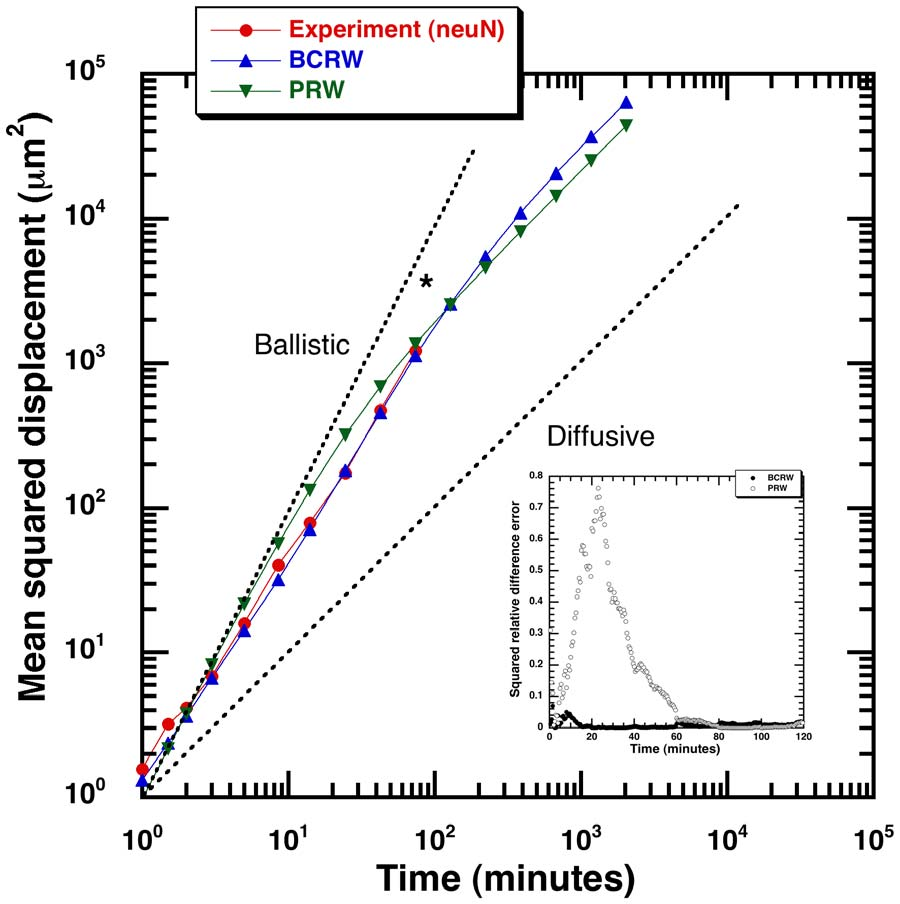
Super-diffusive behavior in mean-squared displacement trends. Simulated mean-squared displacement versus time from a simulation based on BCRW model (blue) compared to the experimental neuN data (red). The bimodal correlation contributes to prolonged super-diffusivity (high persistence) observed in epithelial cells under consideration (neuN cell type). The “*” indicates transition to the diffusive regime in the BCRW model. A fit of the experimental data using a PRW model (green) has been overlaid. *Inset:* Comparison of BCRW and PRW model predictions with the experimental mean-squared displacement. The squared relative difference error (difference normalized using the experimental mean-squared displacement at a given time) for predictions from BCRW and PRW model. The BCRW model predictions are in good agreement with the experiments.

The BCRW model for the epithelial cells differs from a simple correlated random walk such as the often-used PRW model for mammalian cell migration. The BCRW model has different correlations (turn angle distributions) for the directional and re-orientation flights each illustrated in Fig. 5. We performed a simulation based on the proposed BCRW to predict the diffusive properties of such a random walk and compare with the experimental cellular tracks and also with a simple correlated random walk using the PRW model-fit of the experimental data. The results from the simulation in terms of MSD trends are illustrated in Fig. 6. We used mean-squared displacement to line up the two random walk models in order to compare cell-population level predictions of these models using single cell measurements. The mean-squared displacement trends intrinsically include predictions of angle distributions and also the distance traversed during relocation/re-orientation phase in the form of time taken to crossover from ballistic to diffusive regime (i.e, the persistence time).

The flight lengths/net flight lengths of individual human mammary epithelial cells in random motility assays deprived of any directional bias were found to follow exponential statistics (Figs. 3 ad 4). This is in contrast to the recent report of foraging behavior of large animals such as sharks as a whole organism [8] which exhibit Lévy behavior. Our result is, however, consistent with the search strategy in *Dictyostelium* that was reported to be lacking any Lévy statistics [28], [30] and with the revised results on motion of albatrosses [22]. Our result is also similar to the motion of motile flagellated prokaryotic bacterial cells that have Poisson distributed runtimes [33]; given the essentially constant speed with which the bacterial cells move, the run length distribution of bacteria can also be expected to be exponentially distributed.

The PRW model equation (see methods) is based on a velocity-jump process with instantaneous relaxation period [34] which would be true for bacterial migration. Instead, in the BCRW, the re-orientation phase acts as a relaxation period with finite time and has a correlation much smaller than that present in the directional phase (Fig. 5). The re-orientation phase is not completely diffusive and the angle changes during this phase are non-uniform. A cell is not stationary during the re-orientation phase and the total distance traveled during this phase is similar to that traveled during directional phase (Table S1). But the net displacement (Table 1) is higher in the directional phase compared to the re-orientation phase giving rise to higher directionality ratio in this phase [31].

By comparison to the PRW model, the BCRW model can pin-point the factor controlling the directional persistence, either the mean flight lengths (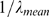) or correlations in the turn angle distribution [7]. The migratory differences in the cell types may exist in differences in the correlations within the different flight types and between the directional flights. The neuT cell type was found to be more persistent than pBabe [31]. The angle change distributions within the directional and re-orientation flights (Fig. 5) for the three cell types confirm a high degree of correlation within directional flights. As the three cell types have similar value of the exponent 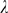, for both the flight types and similar within-flight turn angle distribution; it is the turn angle distribution between the directional flights that seems to control the persistence behavior in a cell type (Fig. 7a in reference [31]).

The 2-hour experimental MSD plot of these cells has a slope greater than 1 indicating prolonged super-diffusive motion (Fig. 12 in reference [31]). A fit of the experimental MSD with a PRW model gives a persistence time of around 10 minutes by creating an early transition to diffusive regime (Fig. 6). This further indicates that the migration strategy adopted by the cells has super-diffusive properties that cannot be attributed to a simple correlated random walk. We find through simulations parameterized by our experiments (neuN cell type), that the BCRW model has super-diffusive properties around experimental time-scale but converges to normal diffusive regime in the long-time limit. A simulated composite correlated random walk incorporating directional and re-orientation phases through the BCRW framework recreates the experimental MSD trend where super-diffusive behavior is maintained over the observed experimental time-scale. We compared the squared relative difference error (see statistical methods) from the PRW model-fit and our BCRW model fit (see *inset* Fig. 6). The BCRW model does a better job in fitting the experimental MSD in the transition regime (10 to 60 minutes) compared to the PRW.

In summary, in this paper we have shown for the first time that the search strategy of mammary epithelial cells (from multi-cellular organisms, humans) in random migration conditions is an intermittent search process (ballistic motile regions (directional) followed by less-persistent search periods (re-orientation)) described by the BCRW model with exponentially distributed flight lengths. The BCRW model provides a new conceptual framework for improved modeling of epithelial cell migration, and was found to fit the experimental data better compared to a simple correlated random walk modeled using the PRW model. This model could predict the observed prolonged super-diffusivity in experimental trajectories, and will form the basis for more realistic simulations of mammalian cell motility with no prior assumptions regarding the diffusive properties of these cells.

## Materials and Methods

### Bimodal Analysis [31] to Identify Directional and Re-Orientation Flights

We use the results of the bimodal analysis [31] method developed by us to analyze the random migration data of three cell types derived from the MCF-10A human mammary epithelial cells, expressing the pBabe vector alone (pBabe), or the normal (neuN) or transforming (neuT) versions of the rat Her 2/Neu oncogene. The migration of cells was followed under random motility conditions without the presence of any externally added chemo-attractant gradients, using time-lapse video-microscopy. The details of the cell culture routine used, cell motility experiments performed and the bimodal analysis technique are elaborated elsewhere [31]. Briefly, the cells were followed for at least two hours in all the experiments and at least five sets of experiments were performed. We use the bimodal analysis results of data collected with a resolution of 40× (1 pixel = 0.163 µm) and a video-microscopy sampling interval of 0.5 minutes. All the three cell types were plated overnight at a low density of approximately 5000 cells per cm^2^ of growth area on tissue-culture plastic. Only cells that were motile for more than two cell diameters (>30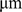), did not adhere to other cells or did not divide or moved out of frame were considered for tracking procedure. The number of cells that were finally filtered for application of bimodal analysis were for pBabe: 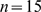, neuN: 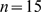 and neuT: 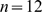.

The bimodal analysis [31] segregates the cellular trajectories of individual mammalian cells, specifically MCF-10A cells (human mammary epithelial cells), into two alternating modes (directional and re-orientation phases) based on a framework similar in spirit to that used in the analysis of bacterial motility [33], [35]. The first step in segregating the directional and re-orientation modes required the determination of the instantaneous direction change, 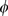, for every time point, *t* of data, i.e., every 0.5 minutes. The values of 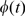 were then compared to an empirically defined cut-off angle, 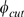, with time points with values of 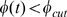 qualifying as directional mode while those with values of 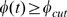 comprising a re-orientation mode. The start of the directional mode was flagged at any time point 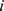 if at least three successive time points have 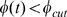, while the beginning of a re-orientation mode was flagged at any time point 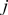 if two successive time points have 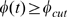. The value for 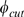 was set to 45°. The angle change distributions for each mode were computed using the 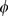 values corresponding to each mode to get an idea of correlation within each mode. An example neuT trajectory with the directional and re-orientation flights is depicted in Fig. 1 (top panel). A directional flight length is the summation of all the consecutive move step-lengths during the directional phase and similarly, a re-orientation flight length is the summation of a series of all the move step-lengths during the re-orientation phase [7]. We define net flight length as the displacement during the given flight.

### Survival Frequency Distributions

The survival frequency of flight lengths is defined as the cumulative frequency of flight lengths greater than any given threshold. For an exponential distribution 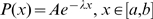; the cumulative distribution function (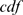) (between the bounds 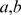) for flight lengths less than any 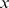 is given by, 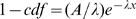. Taking logarithm on both sides, we get,
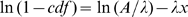
(1)Equation (1) is of the form 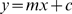, and hence a plot of 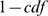 versus 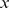 on log-linear scale yields a straight line for an exponential distribution. The slope 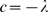, is the inverse of the average value of 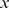. A bin size of 1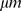 was used to bin the flights.

### Logarithmic Binning of Flights

The presence of Lévy behavior was investigated using the logarithmic binning with normalization method (LBN method) described in the Sims et. al. paper [23]. Logarithmic binning involves increasing the bin sizes in a geometric sequence so that the size of the 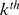 bin is 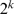 where 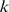 was varied from −2,−1,0,1,2,3,… The normalized frequency was calculated as the ratio of observed frequency to the product of bin width (for a given bin) and number of data points.

### Statistical Analysis

All statistical analysis was done in MATLAB software package (MathWorks, Natick, MA). The Lilliefors test (lillietest.m) revealed that the data were non-paramteric. A two-sample Kolmogorov-Smirnov test (kstest2.m in MATLAB) was used to determine statistical significance between the mean net displacements in the directional (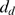) and re-orientation (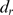) phases for a given cell type. A p-value <0.05 indicates significant difference. Statistical analysis for estimating the fitting parameter for exponential distribution (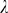) from the survival frequency distributions along with associated 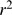 (correlation coefficient) was performed using regstats.m in MATLAB, which also gives the p-values associated with the slopes using t-test. The mean value reported in Tables 1 and S1 for a given cell type is the mean of mean value for each cell (pBabe (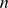 = 15), neuN (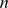 = 15), neuT (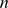 = 12)) in the population while error bars are standard deviations in the means.

The PRW model-fit of the experimental data was performed by fitting the experimental MSD using the following equation: 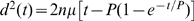, where 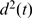 is the MSD, 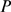 is the persistence time and 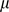 the random motility coefficient, for a system with 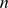 dimensions. The squared relative difference error (RDE) for a given model 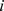 is defined as, 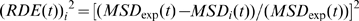, where model 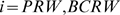
and 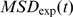 is the experimental MSD at a given time 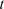.

### *BCRW model*

The BCRW could be thought of as a modified correlated random walk comprising of two alternating modes with varying degree of correlations. This is analogous to an intermittent search strategy having a fast phase oblivious of the presence of any target and a slow responsive, search phase to locate the target [24]. Some of the salient characteristics of this proposed BCRW are: i) flights follow an exponential distribution; ii) move step-lengths comprising the flight are correlated through turn angles randomly drawn from a distribution such as a Gaussian distribution and iii) move step-lengths within the flight are randomly drawn from an exponential distribution.

### BCRW Simulations

The PRW model fit gives a persistence time of around 10 minutes indicating early loss of super-diffusivity unlike the experimental mean-squared displacement. Hence, we performed simulations of the epithelial cell migration based on the proposed BCRW framework to investigate the nature of diffusive properties of such a system in a long time limit. In our simulations for the BCRW exhibited by epithelial cells, exponential flights were generated by sampling an exponential distribution 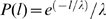 where 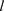 is flight length and 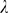 is the mean flight length. In order to generate the flight lengths from the exponential distribution, the inversion method was used, *i.e.*, flight length was generated from the exponential distribution by inserting a uniform random number, 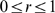, into the inverse of the cumulative distribution of the exponential function, 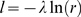. In the BCRW, the 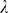 in the directional (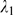) and reorientation (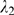) phases can be different. Moreover, because the flight length is of finite size, the maximum flight length, 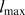, can be defined according to the particular cell type and the occurrence of an undesirably long flight length can be avoided. We also had a minimum cut-off for the flight length, 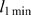 and 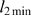, for directional and re-orientation flight, respectively.

The step-lengths in a flight are not straight-line move step-lengths but are correlated through a series of turning angles from a distribution such as the Gaussian distribution 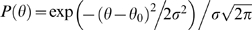 where 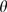 is the turning angle (*i.e.*, deviation from the previous direction), 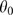 the mean turning angle (in our BCRW simulation 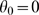), and 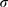 the standard deviation of the distribution. Standard deviation, 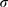, in the Gaussian distribution function determines directionality or persistence of step movement, *i.e.*, smaller 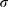 makes the Gaussian distribution narrow and the path more persistent. In the BCRW, two standard deviation values, 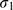 and 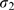, were used in directional and re-orientation flights, respectively. In addition, a correlation between two neighboring directional phases is described by using the Gaussian distribution of turning angles between two directional flights with a standard deviation, 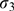.

The step-lengths, 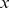, within the flights are randomly drawn from an exponential distribution 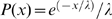 where 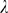 is the mean move step-length in flights. In order to generate the move step-lengths from the exponential distribution, the inversion method was used as before. To test the validity of the BCRW model, experimental mean-squared displacement of the cells is compared with that obtained from the simulation. We find that all the individual step lengths as well as flight lengths in the cellular tracks are exponentially distributed and we use this information to perform a simulation based on proposed BCRW framework.

The turn angles between the individual steps in each flight type is associated with certain degree of persistence or ‘
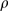
’ parameter associated with a wrapped Cauchy distribution [7]. The turn angle distribution within the directional flights is narrow compared to the spread out re-orientation flight angle distributions (Fig. 5). This implies a higher value of 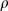 (more persistence) associated with directional flights. We find that a Gaussian distribution provided a better fit to the angle distributions compared to a wrapped Cauchy distribution. A Gaussian distribution has been used earlier to fit the turning angles [36], [37]. We related the standard deviation of the Gaussian distribution to degree of persistence, a higher value indicating lesser persistence. The neuT cell type was found to be more persistent as the turn angle distribution between the directional flights has the least standard deviation compared to pBabes and neuNs. The standard deviation of turn angle distribution in a directional flight is smaller than that of a re-orientation flight. This was incorporated in the BCRW simulations.

### Simulation Parameters

In order to obtain mean-squared displacement of cells from a BCRW simulation, we generated 100 independent trajectories during 2500 minutes, which is not feasible in an experimental situation, however this gives an important insight into the characteristics of cell motility. Parameter values used in the BCRW simulation of neuN cell type are as follows: 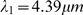, 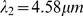, 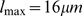, 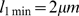, 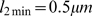 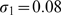, 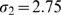, 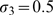 and 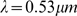.

## Supporting Information

Text S1 Supporting information text file.
(0.04 MB DOC)

Table S1 (0.03 MB DOC)

Table S2 (0.03 MB DOC)

Figure S1 Log-log frequency plots using the logarithmic binning with normalization method along with a fitted exponential function. The logarithmically binned net flight length distributions on log-log scale for the three cell types. Directional net flights lengths are shown in the left panel while re-orientation net flight lengths are on the right. An exponential distribution fitted to the λ (obtained from corresponding survival distribution) is shown in bold curve in black. The fitted exponential distribution is in good agreement with the experimental data points.
(9.41 MB TIF)
