# Supplementary material for: Human Mammary Epithelial Cells Exhibit a Bimodal Correlated Random Walk Pattern
Source: PLoS One. 2010 Mar 10;5(3):e9636. doi: 10.1371/journal.pone.0009636 (PMC2835765; doi:10.1371/journal.pone.0009636)
Supplement: Text S1 — Supporting information text file. (0.04 MB DOC) [file pone.0009636.s001.doc]

**Supplementary Information**

**Human Mammary Epithelial Cells Exhibit A Bimodal Correlated Random Walk Pattern**

Alka A. Potdar^1,2^, Junhwan Jeon^1,2^, Alissa M. Weaver ^2,3^ ,Vito Quaranta^2,3^ and

Peter T. Cummings^1,2,4^

*^1^Department of Chemical and Biomolecular Engineering, Vanderbilt University, Nashville, TN, USA; ^2^Vanderbilt Integrative Cancer Biology Center, Nashville, TN, USA; ^3^Department of Cancer Biology, Vanderbilt University Medical Center, Nashville, TN, USA; ^4^Center for Nanophase Materials Sciences, Oak Ridge National Laboratory, Oak Ridge, TN, USA*

**Supplementary Methods:**

**Maximum likelihood estimates (MLE) and Akaike weights**

We used likelihood estimates and Akaike weights [1,2] to determine which of the two models (exponential or power law) fit our experimental data. The probability density functions for the two models and the corresponding maximum likelihood estimate of the model parameter are as follows [2,3]:

Power law model

(1)

(2)

Exponential model:

(3)

(4)

One parameter was estimated for each of the two models considered (exponential ($\lambda$) and power law ($m$)) using equations 2 and 4. The equations used for calculating Akaike weights are same as the ones detailed in box1 in [2]. Table S2 shows the MLE of parameters for both models along with the Akaike weights for the flight lengths of the three cell types. The exponential model is highly favored in all cases.

**References:**

1. Edwards A (2008) Using likelihood to test for Lévy flight search patterns and for general power-law distributions in nature. Journal of Animal Ecology 77: 1212-1222.

2. Edwards A, Phillips R, Watkins N, Freeman M, Murphy E, et al. (2007) Revisiting Levy flight search patterns of wandering albatrosses, bumblebees and deer. Nature 449: 1044-U1045.

3. Newman MEJ (2005) Power laws, Pareto distributions and Zipf's law. Contemporary Physics 46: 323-351.

4. Bartumeus F, Da Luz MGE, Viswanathan GM, Catalan J (2005) Animal search strategies: A quantitative. random-walk analysis. Ecology 86: 3078-3087.

5. Byers JA (2001) Correlated random walk equations of animal dispersal resolved by simulation. Ecology 82: 1680-1690.

6. Cain ML (1985) Random search by herbivorous insects - A simulation-model. Ecology 66: 876-888.
