# Supplementary material for: Human Mammary Epithelial Cells Exhibit a Bimodal Correlated Random Walk Pattern
Source: PLoS One. 2010 Mar 10;5(3):e9636. doi: 10.1371/journal.pone.0009636 (PMC2835765; doi:10.1371/journal.pone.0009636)
Supplement: Table S1 — (0.03 MB DOC) [file pone.0009636.s002.doc]

|  |  | Cell type |  |
| --- | --- | --- | --- |
|  | pBabe | neuN | neuT |
| $t_{d}$^1,3^ (micron) | 3.43±1.24 | 3.78±1.52 | 4.37±1.99 |
| $t_{r}$^2,3^ (micron) | 4.62±1.73 | 5.76±4.07 | 4.86±1.73 |
| p-values (n^4^) | 0.158 (214) | 0.027 (187) | 0.417 (169) |

**Table S1: Average total distances covered in directional and re-orientation phases.**

Statistical significance to compare between the average total distances traveled in the directional ($t_{d}$) and re-orientation ($t_{r}$) phases using the nonparametric, two-sample Kolmogorov-Smirnov test in MATLAB (kstest2.m). A p-value <0.05 indicates significant difference.

^1^ Mean total distance traveled during directional phase

^2^ Mean total distance traveled during re-orientation phase

^3^ Mean value reported is the mean of mean values for each cell (pBabe (n=15), neuN (n=15), neuT (n=12)) while error bars are standard deviation in the means

^4^ Sample size for the two groups, which is the number of directional or re-orientation phases
