# Supplementary material for: Human Mammary Epithelial Cells Exhibit a Bimodal Correlated Random Walk Pattern
Source: PLoS One. 2010 Mar 10;5(3):e9636. doi: 10.1371/journal.pone.0009636 (PMC2835765; doi:10.1371/journal.pone.0009636)
Supplement: Table S2 — (0.03 MB DOC) [file pone.0009636.s003.doc]

|  | MLE for model parameter | | | Akaike weights | |
| --- | --- | --- | --- | --- | --- |
| Cell type (mode) |  |  |  | Power law | Exponential |
| pBabe (Directional) | 0.322 | 1.5 | 214 | 0 | 1 |
| pBabe (Re-orientation) | 0.237 | 1.468 | 214 | 0 | 1 |
| neuN (Directional) | 0.289 | 1.485 | 187 | 0 | 1 |
| neuN (Re-orientation) | 0.198 | 1.453 | 187 | 0 | 1 |
| neuT (Directional) | 0.237 | 1.455 | 169 | 0 | 1 |
| neuT (Re-orientation) | 0.219 | 1.452 | 169 | 0 | 1 |

**Table S2:** **Maximum likelihood estimates (MLE) of $\lambda$ (parameter for exponential model) and $m$ (parameter for power-law model).**

Maximum likelihood estimates (MLE) of $\lambda$ (parameter for exponential model) and $m$ (parameter for power-law model) using maximum likelihood method and Akaike weights for each model for the flight lengths data of the three cell types. The exponential model is highly favored in all cases as indicated by higher Akaike weights.
