# Supplementary figures and images for: Human Mammary Epithelial Cells Exhibit a Bimodal Correlated Random Walk Pattern
Source: PLoS One. 2010 Mar 10;5(3):e9636. doi: 10.1371/journal.pone.0009636 (PMC2835765; doi:10.1371/journal.pone.0009636)

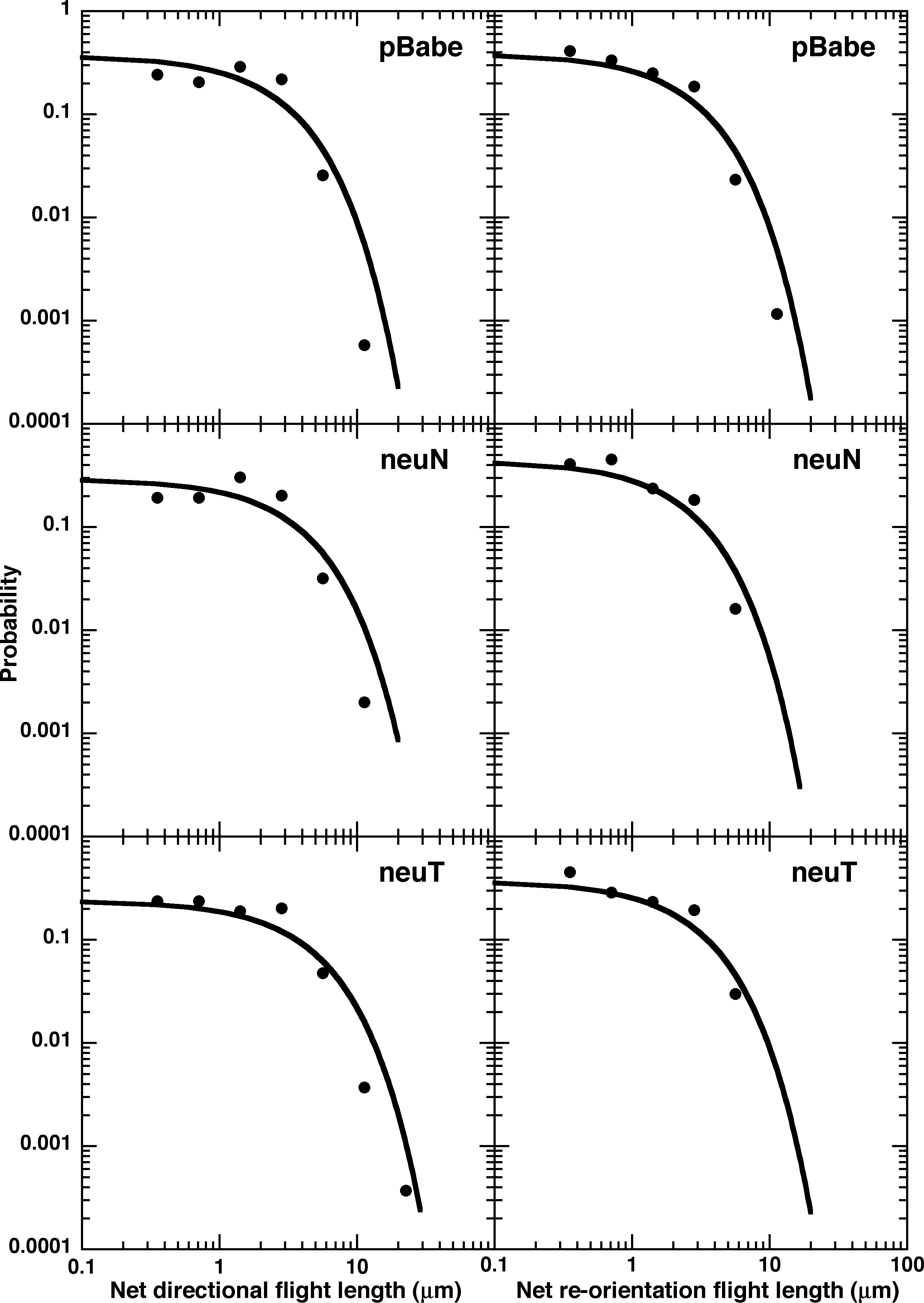

Supplement: Figure S1 — Log-log frequency plots using the logarithmic binning with normalization method along with a fitted exponential function. The logarithmically binned net flight length distributions on log-log scale for the three cell types. Directional net flights lengths are shown in the left panel while re-orientation net flight lengths are on the right. An exponential distribution fitted to the λ (obtained from corresponding survival distribution) is shown in bold curve in black. The fitted exponential distribution is in good agreement with the experimental data points. (9.41 MB TIF) [file pone.0009636.s004.tif]
